# Association of self-reported anxiety and depressive symptoms with incident hypertension among young adults

**DOI:** 10.3389/fcvm.2026.1856400

**Published:** 2026-08-31

**Authors:** Ko-Huan Lin, Chu-Yu Hsu, Yi-Chiung Hsu, Kun-Zhe Tsai, Yun-Chen Chang, Wei-Chun Huang, Xuemei Sui, Carl J. Lavie, Gen-Min Lin

**Affiliations:** 1 Department of Medicine, Hualien Armed Forces General Hospital, Hualien, Taiwan; 2 Psychiatry Division, Hualien Tzu Chi Hospital, Buddhist Tzu Chi Medical Foundation, Hualien, Taiwan; 3 Department of Biomedical Sciences and Engineering, Center for Astronautical Physics and Engineering, National Central University, Taoyuan, Taiwan; 4 Department of Medical Research, Cathay General Hospital, Taipei, Taiwan; 5 Department of Medicine, Taoyuan Armed Forces General Hospital, Taoyuan, Taiwan; 6 Department of Stomatology of Periodontology, Mackay Memorial Hospital, Taipei, Taiwan; 7 Departments of Dentistry, Tri-Service General Hospital, National Defense Medical Center, Taipei, Taiwan; 8 Nursing Department, China Medical University Hospital, Taichung, Taiwan; 9 School of Nursing and Graduate Institute of Nursing, China Medical University, Taichung, Taiwan; 10 Department of Medicine, Pingtung Christian Hospital, Pingtung, Taiwan; 11 Department of Business Management, National Sun Yat-sen University, Kaohsiung, Taiwan; 12 Department of Exercise Science, Arnold School of Public Health, University of South Carolina, Columbia, SC, United States; 13 Ochsner Clinical School, John Ochsner Heart and Vascular Institute, The University of Queensland School of Medicine, New Orleans, LA, United States; 14 Departments of Medicine, Tri-Service General Hospital, National Defense Medical University, Taipei, Taiwan

**Keywords:** anxiety, depression, hypertension, military personnel, young adults

## Abstract

**Aims:**

Previous studies have revealed inconsistent results regarding the association between depressive symptoms and hypertension. Other mood disorders, such as anxiety, usually coexist with depression and require exploration of their impact on depression in patients with hypertension.

**Methods and results:**

This study included 2,448 Taiwanese military personnel who were free of hypertension at baseline. Mood status was assessed using the Brief Symptoms Rating Scale-5 (BSRS-5) score, which includes five domains of depressive symptoms, anxiety, interpersonal sensitivity, insomnia, and hostility, which were evaluated separately using a five-point Likert-type scale of 0–4. Incident hypertension was defined according to the latest American guidelines, and follow-up was conducted from 2014 until the end of 2020. Multivariable Cox regression analysis was used to determine the associations of each mood item and a combination of the significant items with incident hypertension, with adjustments for potential confounders.

**Results:**

There were 125 new-onset stage 2 hypertension cases (5.2%) during a median follow-up period of 6.0 years. The sum of the BSRS-5 scores was not linked to new-onset stage 2 hypertension [hazard ratio (HR) and 95% confidence interval (CI): 1.05 (0.99–1.11)]. Among the five mood domains, depressive symptoms (score ≥1) were significantly associated with new-onset stage 2 hypertension [HR 1.68 (95% CI 1.16–2.45)], whereas anxiety symptoms (score ≥1) showed a borderline association [HR 1.43 (95% CI 0.99–2.08)]. Neither of the associations was linear. The presence of depressive symptoms alone or anxiety symptoms alone (one score ≥1 and another score = 0) was not associated with stage 2 hypertension (HR: 1.11, *p* = 0.71), whereas both scores ≥1 were associated with a higher risk of stage 2 hypertension [HR: 1.71 (95% CI 1.14–2.57)].

**Conclusions:**

Our findings suggest that the coexistence of self-reported anxiety and depressive symptoms may increase the risk of stage 2 hypertension in young military adults.

## Introduction

Depression and hypertension are two prevalent conditions that represent major public health challenges worldwide. Depression affects an estimated 280 million people globally and is a leading cause of disability worldwide (1). Hypertension affects approximately 1.28 billion individuals worldwide and is a major risk factor for cardiovascular disease (CVD), stroke, and renal failure (2).

Despite their distinct clinical presentations, mounting evidence suggests a complex interplay between depression and hypertension. However, findings regarding their association remain inconsistent. Some studies have identified depression as a risk factor for hypertension (3, 4), whereas others have found no association (5) or even reported an inverse relationship (6). These inconsistent findings suggest that additional factors may modify the association between depression and hypertension (7).

Anxiety frequently coexists with depression and may influence the association between depression and hypertension. The combination of depression and other psychiatric conditions, particularly anxiety, is associated with increased mortality in patients with coronary heart disease (8, 9). Although some studies have identified anxiety as a risk factor for hypertension (10, 11), others have reported no association (12, 13) or an inverse relationship (14, 15). More importantly, few studies have specifically examined whether comorbid anxiety modifies the association between depression and hypertension. Although previous studies have investigated depression and anxiety in relation to hypertension, several important knowledge gaps remain. Most studies have evaluated depression or anxiety separately, making it difficult to determine whether coexisting anxiety modifies the association between depression and hypertension. In addition, evidence from relatively young and otherwise healthy populations remains limited, as most previous studies have been conducted in community-based or older populations with substantial cardiometabolic comorbidities. Hildrum et al. reported that depression and anxiety are associated with low BP and that comorbid anxiety further amplifies this association (14). Therefore, the present study aimed to investigate the association between depressive symptoms and hypertension while further examining the potential modulatory effect of comorbid anxiety symptoms in a relatively young military cohort. This study seeks to provide additional evidence on the independent and combined associations of depressive and anxiety symptoms with hypertension by evaluating these symptoms simultaneously in a homogeneous population with fewer age-related comorbidities.

## Methods

### Study population

The study cohort included 4,080 military men and women aged 18–50 years who were not taking lipid-lowering medications or antihypertensives from the cardiorespiratory fitness and health in eastern armed forces (CHIEF) study in Hualien, Taiwan, in 2014 (16). Although the CHIEF cohort enrolled military personnel aged 18–50 years, the present study specifically focused on young military personnel. Therefore, participants aged ≥40 years were excluded from the analysis to reduce the potential confounding effect of age-related hypertension.

The aim of this cohort study was to clarify the correlates and associations between physical activity (PA) levels and cardiometabolic disorders. The CHIEF study has been described in detail previously (16–18). The study design and protocol have been approved by the Institutional Review Board (IRB) of the Mennonite Christian Hospital (No. 16-05-008) in Hualien, Taiwan. Written informed consent was obtained from all the participants.

### Annual health examinations (2014–2020)

Personal and medical history of each participant was self-reported in response to a set of printed questions. The status (current active vs. former/never) of betel nut chewing, cigarette smoking, and alcohol consumption, as well as moderate-intensity PA, which was assessed by running hours per week (<150, 150–299, and ≥300), according to the 2018 American guideline (19) in the past 6 months, was self-reported at baseline (20, 21).

Each participant's body height, body weight, and waist circumference were measured in the standing position. The definition of body mass index (BMI) was defined as the body weight (kg) divided by the body height squared (m^2^). BP of each participant was measured once over the right arm in the sitting position using an automatic device (FT201 Parama-Tech Co., Ltd, Fukuoka, Japan) with the oscillometric method (22, 23). If the systolic BP level ≥130 mmHg or diastolic BP ≥80 mmHg was obtained initially, a second BP measurement was requested after a rest period of 15 min or more. The final BP value was the average of the initial and second BP readings. Plasma concentrations of blood urea nitrogen (BUN), creatinine, and serum uric acid (SUA) were measured from an overnight fasting blood sample of each participant using an auto analyzer (Olympus AU640, Kobe, Japan). The estimated glomerular filtration rate (eGFR) was calculated using the 2021 Chronic Kidney Disease Epidemiology Collaboration (CKD-EPI) equation (24).

### Mental health assessment

Mental health was evaluated using the five-item Brief Symptom Rating Scale (BSRS-5), in which each participant self-reported their psychological stress status in the past 3 months (25, 26). The BSRS-5 score consists of feeling tense (anxiety symptoms), feeling low in mood (depressive symptoms), trouble falling asleep (insomnia), feeling inferior to others (interpersonal sensitivity), and feeling easily annoyed or irritated (hostility). Each domain of the BSRS-5 included a five-point Likert-type scale for scoring, where 0 = not at all, 1 = mild, 2 = moderate, 3 = severe, and 4 = extremely severe. A score of ≥1 for each BSRS-5 symptom domain was used to indicate the presence of a symptom, whereas a score of 0 indicated its absence. The presence of mental distress was defined as a BSRS-5 score >6. The internal consistency (Cronbach’s alpha) coefficients ranged from 0.77 to 0.90, and the test–retest reliability coefficient score was 0.82 (25).

### Definition of hypertension

According to the 2017 American College of Cardiology (ACC)/American Heart Association (AHA) guidelines (27), stage 1 hypertension was defined as systolic BP of 130–139 mmHg and/or diastolic BP of 80–89 mmHg, and/or the use of antihypertensive medication. Stage 2 hypertension was defined as a systolic BP of 140–159 mmHg and/or diastolic BP of 90–109 mmHg, and/or the use of antihypertensive medication. Isolated systolic hypertension (ISH) is defined as when the systolic BP level meets the hypertension criteria, whereas the diastolic BP level does not. Isolated diastolic hypertension (IDH) is defined when the systolic BP level does not meet the hypertension criteria, but the diastolic BP does. Combined hypertension (CH) was diagnosed if both systolic BP and diastolic BP met the hypertension criteria.

### Statistical analysis

Baseline characteristics of the participants were presented as mean ± standard deviation (SD)for continuous variables and numbers (percentage) for categorical variables. Each participant was followed up from baseline (2014) until the first occurrence of new-onset hypertension, loss to follow-up, or the end of follow-up (31 December 2020). The proportional hazards assumption was assessed using the Supremum test (*p* = 0.36). Multivariable Cox regression analysis was used to determine the association of BSRS-5 scores (with each 1 score increase) with incident hypertension (any), with simultaneous adjusting for sex, age, baseline systolic BP, diastolic BP, BMI, alcohol consumption, tobacco smoking status, betel chewing status, SUA, BUN, eGFR, and PA. The associations of BSRS-5 score >6, an anxiety score ≥1, a depression score ≥1, an insomnia score ≥1, interpersonal sensitivity ≥1, and a hostility score ≥1 with incident stage 1 and stage 2 hypertension were also examined, respectively. These covariates were selected in the Cox Models based on the established associations for hypertension and mental stress in prior studies (18, 26, 28). To examine the linearity between the severity of anxiety and depressive symptoms and hypertension, the psychiatric symptoms were separately stratified by their scores of  0, 1, and ≥2, and their corresponding associations with hypertension were examined. In addition, to examine the interplay of anxiety and depressive symptoms in reation to incident hypertension, the study participants were grouped as follows: those with an anxiety score of  0 and depression score  of 0 (neither, the reference group), those with an anxiety score of ≥1/depression score of  0, or an anxiety score  of  0/depression score of ≥1 (either), and those with an anxiety score of ≥1/depression score ≥1 (both). The associations of the three groups with stage 1 and stage 2 hypertension (any, ISH, IDH, and CH) are examined, respectively. Subgroup analyses for the association between the anxiety score ≥1/depression score ≥1 group with stage 1 and stage 2 hypertension within age (<30 years vs. ≥30 years) and BMI (<25.0 kg/m^2^ vs. ≥25.0 kg/m^2^) and formal testing for interactions were performed. A *P*-value <0.05 was considered significant. All statistical analyses were performed using SPSS v25.0 for Windows (IBM Corp., Armonk, NY, USA).

## Results

In this cohort study, participants with baseline hypertension (*N* = 1,024), those who moved out of the Hualien or Taitung military bases and were lost to follow-up (*N* = 550), and those aged ≥40 years (*N* = 58) were excluded from the initial cohort, leaving a final sample of 2,448 subjects for analysis. Over a median follow-up period of 6.0 years, 784 participants (32.5%) developed stage 1 hypertension, and 125 participants (5.2%) developed stage 2 hypertension.

The baseline characteristics of the study cohort are shown in Table 1. The analyzed cohort comprised 2,448 military personnel with an average age of 28.15 ± 5.65 years and a male predominance of 87.9%. The mean total BSRS-5 score was 1.9 ± 2.71, and the scores in different symptom domains were listed separately. The baseline systolic BP and diastolic BP were 112.10 ± 10.31 and 66.50 ± 7.08 mmHg, respectively.

**Table 1:** Baseline clinical characteristics of the study population.

Variables	*N* = 2,448
BSRS-5 score	1.90 ± 2.71
Insomnia score	0.48 ± 0.73
Anxiety score	0.38 ± 0.62
Hostility score	0.43 ± 0.67
Depression score	0.35 ± 0.62
Interpersonal sensitivity score	0.27 ± 0.57
Age (years)	28.15 ± 5.65
Male sex (%)	2,123 (87.9)
Substance use status (%)	
Alcohol drinking, active	960 (39.8)
Betel nut chewing, active	237 (9.8)
Cigarette smoking, active	867 (35.9)
Physical activity levels (%)	
<150 min/week	525 (21.7)
150–299 min/week	930 (38.5)
≥300 min/week	960 (39.8)
Systolic blood pressure (mmHg)	112.10 ± 10.31
Diastolic blood pressure (mmHg)	66.50 ± 7.08
BMI (kg/m^2^)	24.26 ± 3.08
Waist circumference (cm)	81.49 ± 8.29
Blood test	
Total cholesterol (mg/dL)	172.01 ± 33.05
LDL-C (mg/dL)	103.62 ± 29.10
HDL-C (mg/dL)	49.06 ± 10.32
Triglycerides (mg/dL)	102.55 ± 69.81
Fasting glucose (mg/dL)	92.67 ± 12.78
Uric acid (mg/dL)	6.43 ± 1.36
BUN (mg/dL)	12.61 ± 2.87
eGFR (mL/min/1.73m^2^)	101.40 ± 14.91

BMI, body mass index; BUN, blood urea nitrogen; BSRS-5, Brief Symptoms Rating Scale-5, eGFR, estimated glomerular filtration rate; HDL-C, high-density lipoprotein cholesterol; LDL-C, low-density lipoprotein cholesterol.

The multivariate Cox regression analysis of BSRS-5 scores with the incidence of hypertension is shown in Table 2. There was no significant association between incident hypertension (stages 1 and 2) and BSRS-5 scores. Among the five symptoms in BSRS-5, the presence of depressive symptoms (score ≥1) was significantly associated with incident stage 2 hypertension [hazard ratio (HR): 1.68%; 95% confidence interval (CI): 1.16–2.45]. Anxiety symptoms showed a borderline association with incident stage 2 hypertension (HR 1.43, 95% CI 0.99–2.08; *p* = 0.06). As shown in Supplementary Table S1, the presence of depressive symptoms (score ≥1) was significantly associated with incident stage 2 IDH [HR 1.67 (95% CI 1.08–2.59)] and showed a borderline association with stage 2 ISH [HR 2.04 (95% CI 0.98–4.25), *p* = 0.06]. Anxiety symptoms (score ≥1) also showed a borderline association with incident stage 2 IDH [HR 1.49 (95% CI 0.96–2.29), *p* = 0.07].

**Table 2:** Multivariable Cox regression analysis of mental stress and incident hypertension.

Variables	Stage 1 hypertension		Stage 2 hypertension	
	HR (95% CI)	*p*-Value	HR (95% CI)	*p*-Value
BSRS-5 score (continuous variables)	1.01 (0.99–1.04)	0.29	1.05 (0.99–1.11)	0.10
BSRS-score ≥6	1.00 (0.84–1.19)	0.97	1.24 (0.71–2.15)	0.45
Insomnia score ≥1	1.01 (0.88–1.16)	0.88	1.04 (0.72–1.52)	0.82
Anxiety score ≥1	1.09 (0.94–1.26)	0.24	1.43 (0.99–2.08)	0.06
Hostility score ≥1	1.04 (0.90–1.20)	0.60	1.28 (0.88–1.87)	0.19
Depression score ≥1	1.12 (0.97–1.29)	0.12	1.68 (1.16–2.45)	0.006
Interpersonal sensitivity score ≥1	1.06 (0.90–1.24)	0.48	1.19 (0.79–1.80)	0.40

Data are presented as hazard ratios (HR) and 95% confidence intervals (CI) using multivariable Cox regression analysis adjusted for age, sex, baseline systolic blood pressure, baseline diastolic blood pressure, substance use status, physical activity, body mass index, serum uric acid, blood urea nitrogen, and estimated glomerular filtration rate. BSRS-5 indicates Brief Symptoms Rating Scale-5.

 The results of Cox regression analysis for depressive and anxiety symptoms with the incidence of hypertension are shown in Tables 3, 4. In Table 3, it is evident that the associations of depressive and anxiety symptoms with incident hypertension were not linear. The incidence of stage 2 hypertension with a depression score of  1 was greater than with a depression score of ≥2 [HRs: 1.74 (95% CI 1.17–2.59) and 1.48 (95% CI 0.73–3.01), respectively]. Similarly, an anxiety score of  1 was greater than an anxiety score of ≥2 [HRs: 1.49 (95% CI 1.00–2.21) and 1.21 (95% CI 0.57–2.56), respectively]. As shown in Table 4, having either depressive or anxiety symptoms alone (mutually exclusive) was not significantly associated with incident hypertension [HRs 1.13 (95% CI 0.93–1.37) for stage 1 hypertension and 1.11 (95% CI 0.64–1.92) for stage 2 hypertension, respectively]. However, the presence of both depressive and anxiety symptoms was significantly associated with stage 2 hypertension [HR: 1.71 (95% CI 1.14–2.57), *p* = 0.009].

**Table 3:** Multivariable cox regression analysis of depressive mood levels with incident hypertension.

Variables	Stage 1 hypertension		Stage 2 hypertension	
	HR (95% CI)	*p*-Value	HR (95% CI)	*p*-Value
Depression				
0	1.00		1.00	
1	1.12 (0.96–1.31)	0.16	1.74 (1.17–2.59)	0.007
≥2	1.13 (0.86–1.48)	0.38	1.48 (0.73–3.01)	0.27
*p*-Value for trend		0.14		0.01
Anxiety				
0	1.00		1.00	
1	1.11 (0.96–1.29)	0.17	1.49 (1.00–2.21)	0.04
≥2	0.99 (0.74–1.32)	0.94	1.21 (0.57–2.56)	0.62
*p*-Value for trend		0.43		0.12

Data are presented as hazard ratios (HR) and 95% confidence intervals (CI) using multiple Cox regression analysis adjusted for age, sex, baseline SBP, baseline DBP, substance use disorder, PA levels, BMI, total cholesterol, uric acid, BUN, and eGFR.

**Table 4:** Multivariable cox regression analysis of depressive and anxiety mood status with incident hypertension.

Depression scores ≥1 and anxiety scores ≥1			Stage 1 hypertension			Stage 2 hypertension	
	*N*	Events	HR (95% CI)	*p*-Value	Events	HR (95% CI)	*p*-Value
Neither (reference)	1,541	558	1.00		69	1.00	
Only one	344	133	1.13 (0.93–1.37)	0.21	16	1.11 (0.64–1.92)	0.71
Both	555	224	1.12 (0.95–1.31)	0.18	40	1.71 (1.14–2.57)	0.009
*p*-Value for trend				0.14			0.01

Data are presented as hazard ratios (HR) and 95% confidence intervals (CI) using multivariable Cox regression analysis adjusted for age, sex, baseline systolic blood pressure, baseline diastolic blood pressure, substance use status, physical activity, body mass index, serum uric acid, blood urea nitrogen, and estimated glomerular filtration rate.

Separate analyses of depressive and anxiety symptoms with the incidence of ISH, IDH, and CH are shown in Table 5. The combination of both symptoms was significantly associated with stage 2 IDH (HR 1.76, 95% CI 1.10–2.81), whereas the association with stage 1 IDH did not reach statistical significance (HR 1.27, 95% CI 0.98–1.25). Subgroup analyses of the influence of age and BMI on the association between combined depression and anxiety symptoms and incident hypertension are shown in Supplementary Table S2. A BMI of <25 kg/m^2^ was significantly associated with incident stage 2 hypertension [HR: 1.92 (95% CI 1.02–3.60)]. The association between age ≥30 years and incident stage 2 hypertension is borderline significant [HR: 1.64 (0.99–2.73), *p* = 0.06]. *p*-Values for the interaction within both subgroups did not reach significance.

**Table 5:** Multivariable cox regression analysis of depressive and anxiety mood with incident hypertension.

Depression scores ≥1 and anxiety scores ≥1			ISH			IDH			CH	
	*N*	Events	HR (95% CI)	*p*-Value	Events	HR (95% CI)	*p*-Value	Events	HR (95% CI)	*p*-Value
Stage 1 hypertension										
Neither (reference)	1,541	216	1.00		173	1.00		169	1.00	
Either one	344	33	0.87 (0.60–1.24)	0.44	57	1.39 (1.02–1.89)	0.03	43	1.22 (0.87–1.72)	0.25
Both	555	82	1.27 (0.98–1.65)	0.07	69	1.11 (0.83–1.48)	0.49	73	1.27 (0.95–1.69)	0.10
Stage 2 hypertension										
Neither (reference)	1,541	216	1.00		173	1.00		169	1.00	
Either one	16	5	1.70 (0.61–4.74)	0.30	11	1.02 (0.53–1.97)	0.95	2	0.58 (0.13–2.58)	0.47
Both	40	10	1.88 (0.83–4.26)	0.13	30	1.76 (1.10–2.81)	0.01	5	0.97 (0.34–2.80)	0.96

Data are presented as hazard ratios (HR) and 95% confidence intervals (CI) using multivariable Cox regression analysis adjusted for age, sex, baseline systolic blood pressure, baseline diastolic blood pressure, substance use status, physical activity, body mass index, serum uric acid, blood urea nitrogen, and estimated glomerular filtration rate. ISH, isolated systolic hypertension; IDH, isolated diastolic hypertension; CH, combined hypertension.

## Discussion

In this study, we found that depressive symptoms were associated with incident stage 2 hypertension, and this association was not dose-dependent, indicating the presence of confounding factors such as anxiety symptoms. Meanwhile, we noted that anxiety symptoms tended to be associated with incident stage 2 hypertension. In the following analysis, we found that the association became much more pronounced if depressive symptoms were accompanied by anxiety symptoms, indicating that there was a mutual and synergistic effect of anxiety and depressive symptoms on the development of stage 2 hypertension. Interestingly, this association appeared to be stronger among participants with mild symptoms than among those with more severe symptoms. One possible explanation is the relatively small number of participants in the higher symptom categories, which may have reduced the statistical power and resulted in less precise estimates. Therefore, these findings should be interpreted with caution.

There is substantial literature addressing the relationship between depression and hypertension. However, the findings are conflicting, with some studies suggesting a positive correlation between depression and hypertension, while others do not. A prospective cohort study, including 1,920 subjects, found a marginally increased risk of hypertension among subjects with depression [odds ratio (OR) = 2.16, 95% CI 0.94–4.98] and a significantly increased risk of hypertension incidence within 1 year of the onset of depression (OR = 3.67, 95% CI: 1.25–10.79) (29). A Canadian national health survey, including 115,071 subjects at a median age of 64 years, showed that major depressive disorder is associated with self-reported diagnosis of hypertension. This association is even more pronounced in the younger group compared to those older than 64 years (OR =  2.8, 95% CI: 2.5–3.1 vs. OR =  1.2, 95% CI: 1.1–1.3) (4). A Taiwanese nationwide health insurance database, including 766,725 subjects, revealed a significantly higher annual incidence of hypertension in patients with major depression compared to the general population [3.96% vs. 2.90%, risk ratio (RR) = 1.19, 95% CI 1.08–1.31]. It also reported a more pronounced risk in the younger population (RR = 1.7, 95% CI: 1.28–2.24 for ages 18–39; RR = 1.12, 95% CI: 0.97–1.29 for ages 40–59; RR = 1.16, 95% CI 1.00–1.35 for ages ≥60) (30).

However, a Brazilian study involving 1,174 subjects aged 18–80 years found that lifetime major depression was not associated with hypertension (BP ≥ 140/90 mmHg) in a bivariate analysis (RR = 1.15, 95% CI: 0.75–1.76) (5). In contrast, a cross-sectional study comprising 590 controls and 2,028 patients with major depressive disorder or anxiety disorders at a mean age of approximately 40 years, found that remitted and currently depressed subjects had significantly lower mean systolic BP (*β* = −1.74, *P* = 0.04 and *β* = −2.35, *P* = 0.004, respectively) and also had significantly less chance of ISH than normal controls (31). They also observed that diastolic BP was significantly higher in subjects with anxiety disorders; however, anxiety and incident hypertension were not significantly related. A prospective Norway cohort study comprising 36,530 subjects with a mean age of 44.3 years, followed for 11 years, found that low systolic BP (<10th percentile) was associated with pooled scores of depression and anxiety (OR = 1.30, 95% CI: 1.08–1.57), and changes in depressive and anxiety symptoms were inversely correlated with systolic BP levels (6). Separate analyses of individual depression and anxiety items found a consistent, inverse association with systolic BP. Further cross-sectional analysis also revealed an inverse relationship between depression/anxiety and systolic BP. There was no significant pattern in the analysis of diastolic BP.

According to two previous studies (4, 30), the association between depression and hypertension was shown to be more significant in the younger age group. Our study, based on a Taiwanese military cohort that is relatively younger (aged 28.15 ± 5.65 years) than those in other studies, also supports the positive relationship between depressive symptoms and hypertension. One possible explanation is that depressive symptoms may be associated with increased arterial stiffness, which has been linked to elevated blood pressure in previous studies. However, arterial stiffness was not measured in the present study, and this mechanism remains speculative (32, 33). Another possible explanation is that individuals with depressive symptoms may be more likely to engage in unhealthy lifestyle behaviors, which have been associated with hypertension in previous studies. However, these lifestyle factors were not directly assessed in this study.

According to a meta-analysis of cohort studies (34), anxiety was identified as an independent risk factor for hypertension (OR =  1.18, 95% CI: 1.02–1.37 in 13 cross-sectional studies; HR = 1.55, 95% CI: 1.24–1.94 in eight longitudinal studies). Our study suggests that anxiety symptoms show a borderline association with incident hypertension and may strengthen the association between depressive symptoms and hypertension. Depressive and anxiety symptoms are highly comorbid. The literature shows that 45.7% of patients with major depression may have one or more anxiety disorders in their lifetime, and lifetime comorbidity with depression in social anxiety disorder, panic disorder, and generalized anxiety may reach 20%–70%, 50%, and 43%, respectively (35). Also, anxiety and depression share a common pathway in the modulation of BP, such as the renin–angiotensin system (36), sympathetic activation (37), and vascular resistance (38). In addition, endothelial dysfunction with reduced nitric oxide bioavailability has been proposed as another potential mechanism linking depressive and anxiety symptoms to hypertension by increasing the vascular tone and impairing vasodilation (39). Chronic low-grade inflammation may also contribute to this association, as inflammatory cytokines promote endothelial dysfunction, increase vascular resistance, and facilitate the development of hypertension (40). Notably, workload-related anxiety, which is much more prevalent in young adults than in their older counterparts, plays an important role in the incidence of hypertension (41). This may support our findings that anxiety symptoms might enhance the positive correlation between depressive symptoms and BP, especially among young adults. As mentioned earlier, the combination of depression and other comorbid psychiatric conditions, such as anxiety, has additive effects on mortality risk in patients with CVD (8, 9).

Our study had several limitations. First, the cohort population was confined to military personnel, which may limit the generalizability of the findings to the broader civilian population. Military personnel often experience unique stressors, training regimens, and healthcare systems that may not be representative of the general public. In addition, the homogeneity of the military cohort may mask the variability that could be present in a more diverse population. Second, our study is male-predominant (88%), which is the nature of the Taiwanese military composition, and this may further hamper generalizability. Moreover, because the cohort consisted predominantly of young Taiwanese military personnel, caution is warranted when extrapolating these findings to older adults, women, and non-Taiwanese populations. Third, several potentially important confounding factors were not available in the present study and, therefore, could not be included in the analyses. These include family history of hypertension, dietary sodium intake, sleep disorders, socioeconomic status, and use of psychotropic medications, all of which may influence blood pressure or are associated with depressive and anxiety symptoms. Consequently, residual confounding cannot be excluded completely. Future studies incorporating these variables are warranted to further clarify the observed associations. Fourth, the BSRS-5 is a screening instrument rather than a diagnostic tool; therefore, misclassification of depression and anxiety statuses cannot be excluded. Fifth, blood pressure was measured during annual health examinations rather than through ambulatory or home blood pressure monitoring. Therefore, some participants may have been misclassified because of white-coat or masked hypertension, which should be considered when interpreting our findings.

## Conclusion

Our study findings support that depressive symptoms are associated with stage 2 hypertension and that the presence of anxiety symptoms may amplify this association. These findings underscore the importance of considering psychological factors when assessing and treating hypertension. Future studies should further explore these interactions to develop more comprehensive approaches to hypertension prevention and management that address both physical and mental health (42, 43).

## Data Availability

The raw data supporting the conclusions of this article will be made available by the authors, without undue reservation.
