# Supplementary material for: Association of self-reported anxiety and depressive symptoms with incident hypertension among young adults
Source: Front Cardiovasc Med. 2026 Aug 31;13:1856400. doi: 10.3389/fcvm.2026.1856400 (PMC13572620; doi:10.3389/fcvm.2026.1856400)
Supplement: Supplementary file 1 [file Table1.docx]

Supplemental Table 1. Multivariable Cox Regression Analysis of the Presence of Depressive Symptom and Anxiety Symptom With Incidences of Specific Hypertension

| Depression and anxiety | ISH |  |  | IDH |  |  | CH |  |
| --- | --- | --- | --- | --- | --- | --- | --- | --- |
|  | HR (95% CI) | P value |  | HR (95% CI) | P value |  | HR (95% CI) | P value |
| Depression |  |  |  |  |  |  |  |  |
| Stage 1 hypertension | 1.19 (0.93 – 1.52) | 0.15 |  | 1.13 (0.87 – 1.45) | 0.36 |  | 1.30 (1.00 – 1.68) | 0.05 |
| Stage 2 hypertension | 2.04 (0.98 – 4.25) | 0.06 |  | 1.67 (1.08 – 2.59) | 0.02 |  | 0.82 (0.29 – 2.29) | 0.70 |
| Anxiety |  |  |  |  |  |  |  |  |
| Stage 1 hypertension | 1.19 (0.93 – 1.51) | 0.16 |  | 1.13 (0.88 – 1.41) | 0.35 |  | 1.16 (0.90 – 1.50) | 0.25 |
| Stage 2 hypertension | 1.49 (0.71 – 3.15) | 0.29 |  | 1.49 (0.96 – 2.29) | 0.07 |  | 1.02 (0.40 – 2.62) | 0.96 |

Data are presented as hazard ratios (HR) and 95% confidence intervals (CI) using multiple cox regression analysis with age, sex, baseline systolic blood pressure, baseline diastolic blood pressure, substance use status, physical activity, body mass index, serum uric acid, blood urea nitrogen and estimated glomerular filtration rate adjustments.

Abbreviations: ISH, isolated systolic hypertension; IDH, isolated diastolic hypertension; CH, combined hypertension.

**Supplemental Table 2.** Subgroup Analysis for the Association of Depression score ≥1/Anxiety score ≥1 With Incident Stage 1 Hypertension and Stage 2 Hypertension

|  |  | Stage I Hypertension | | |  | Stage 2 hypertension | | |
| --- | --- | --- | --- | --- | --- | --- | --- | --- |
|  | N | HR (95% CI) | P value | P value for interaction |  | HR (95% CI) | P value | P value for interaction |
| Age |  |  |  |  |  |  |  |  |
| <30 years | 1322 | 1.11 (0.85 – 1.45) | 0.34 | 0.61 |  | 1.57 (0.77 – 3.19) | 0.21 | 0.95 |
| ≥30 years | 1118 | 1.12 (0.91 – 1.37) | 0.27 |  |  | 1.64 (0.99 – 2.73) | 0.06 |  |
| Body mass index |  |  |  |  |  |  |  |  |
| <25 kg/m^2^ | 1485 | 1.06 (0.84 – 1.35) | 0.62 | 0.53 |  | 1.92 (1.02 – 3.60) | 0.04 | 0.72 |
| ≥25 kg/m^2^ | 955 | 1.17 (0.94 – 1.45) | 0.17 |  |  | 1.43 (0.84 – 2.46) | 0.19 |  |

Data are presented as hazard ratios (HR) and 95% confidence intervals (CI) using multivariable Cox regression analysis with age, sex, baseline systolic blood pressure, baseline diastolic blood pressure, substance use status, physical activity, body mass index, serum uric acid, blood urea nitrogen and estimated glomerular filtration rate adjustments.
